# Application of the Gradient-Boosting with Regression Trees to Predict the Coefficient of Friction on Drawbead in Sheet Metal Forming

**DOI:** 10.3390/ma17184540

**Published:** 2024-09-15

**Authors:** Sherwan Mohammed Najm, Tomasz Trzepieciński, Salah Eddine Laouini, Marek Kowalik, Romuald Fejkiel, Rafał Kowalik

**Affiliations:** 1 Kirkuk Technical Engineering College, Northern Technical University, Kirkuk 36001, Iraq; sherwan.mohammed@gpk.bme.hu; 2 Department of Manufacturing Science and Engineering, Budapest University of Technology and Economics, Műegyetemrkp 3, 1111 Budapest, Hungary; 3 Department of Manufacturing Processes and Production Engineering, Rzeszow University of Technology, al. Powstancow Warszawy 8, 35-959 Rzeszów, Poland; 4 Department of Process Engineering, Faculty of Technology, University of El Oued, El-Oued 39000, Algeria; salaheddine-laouini@univ-eloued.dz; 5 Laboratory of Biotechnology Biomaterial and Condensed Matter, Faculty of Technology, University of El Oued, El-Oued 39000, Algeria; 6 Faculty of Mechanical Engineering, Casimir Pulaski Radom University Radom, 54 Stasieckiego Street, 26-600 Radom, Poland; m.kowalik@uthrad.pl; 7 Department of Mechanics and Machine Building, The University College of Applied Sciences in Krosno, ul. Wyspiańskiego 20, 38-400 Krosno, Poland; romuald.fejkiel@pans.krosno.pl; 8 Institute of Technical Sciences and Aeronautics, The University College of Applied Sciences in Chełm, ul. Pocztowa 54, 22-100 Chełm, Poland; rk16410@nauka.panschelm.edu.pl

**Keywords:** coefficient of friction, drawbead, drawpiece, forming parameters, friction, metal forming

## Abstract

Correct design of the sheet metal forming process requires knowledge of the friction phenomenon occurring in various areas of the drawpiece. Additionally, the friction at the drawbead is decisive to ensure that the sheet flows in the desired direction. This article presents the results of experimental tests enabling the determination of the coefficient of friction at the drawbead and using a specially designed tribometer. The test material was a DC04 carbon steel sheet. The tests were carried out for different orientations of the samples in relation to the sheet rolling direction, different drawbead heights, different lubrication conditions and different average roughnesses of the countersamples. According to the aim of this work, the Features Importance analysis, conducted using the Gradient-Boosted Regression Trees algorithm, was used to find the influence of several parameter features on the coefficient of friction. The advantage of gradient-boosted decision trees is their ability to analyze complex relationships in the data and protect against overfitting. Another advantage is that there is no need for prior data processing. According to the best of the authors’ knowledge, the effectiveness of gradient-boosted decision trees in analyzing the friction occurring in the drawbead in sheet metal forming has not been previously studied. To improve the accuracy of the model, five MinLeafs were applied to the regression tree, together with 500 ensembles utilized for learning the previously learned nodes, noting that the MinLeaf indicates the minimum number of leaf node observations. The least-squares-boosting technique, often known as LSBoost, is used to train a group of regression trees. Features Importance analysis has shown that the friction conditions (dry friction of lubricated conditions) had the most significant influence on the coefficient of friction, at 56.98%, followed by the drawbead height, at 23.41%, and the sample width, at 11.95%. The average surface roughness of rollers and sample orientation have the smallest impact on the value of the coefficient of friction at 6.09% and 1.57%, respectively. The dispersion and deviation observed for the testing dataset from the experimental data indicate the model’s ability to predict the values of the coefficient of friction at a coefficient of determination of *R*^2^ = 0.972 and a mean-squared error of *MSE* = 0.000048. It was qualitatively found that in order to ensure the optimal (the lowest) coefficient of friction, it is necessary to control the friction conditions (use of lubricant) and the drawbead height.

## 1. Introduction

In the modern automotive industry, sheet metal forming processes are widely used to produce components with complex shapes [1,2]. The sheet metal forming process involves many challenges and factors that must be taken into account at the drawpiece design stage. The possibility of forming sheets in the deep drawing process is related to their mechanical properties (yield strength, elongation, formability) [3,4], processing conditions (temperature) [5,6] and lubrication conditions [7,8]. Friction is generally undesirable during metal working processes. Friction is a phenomenon that occurs when one body begins to slide against the other. The complexity of this phenomenon results from many factors that change during contact between workpiece and tools in sheet forming processes [9]. These factors include the surface roughness of the friction pair [10,11], the type of anti-wear coatings on the tool surface [12], the type of contact (dynamic or static) [13] and the properties of lubricant used [14]. In the case of drawpieces with complex shapes, undesirable wrinkling of the sheet metal surface or uncontrolled flow of the sheet material in the processing zone may occur [15]. The basic material for the production of car bodies and load-bearing elements in motor vehicles are carbon steel sheets [16], including high-strength steels [17,18,19]. These are sheets with very good formability and strength; they also have a very good tendency to work hardening [20]. The basic way to reduce friction in sheet forming processes is to use an appropriate lubricant [21]. The task of the lubricant is to provide a continuous layer separating the friction surfaces, and the lubricant should be adapted to the contact pressure and temperature in the contact zone. The effectiveness of the lubricant application also depends on the roughness of the surfaces of the cooperating bodies [22]. Surface roughness is closely related to the formation of open or closed oil pockets that retain the lubricant during the mutual movement of the friction pair surfaces [23]. In cold sheet metal forming processes, the use of liquid lubricants dominates. At elevated temperatures, solid lubricants containing primarily MoS_2_ are used more often. In addition to special lubricants adapted to stamping operations, the following lubricants, intended for other applications, are usually used in sheet metal forming: machine oils, engine oils, gear oils, etc. Recently, research has also been developing on the use of vegetable oil-based lubricants belonging to the group of biodegradable substances. They are generally less toxic, more environmentally friendly and reduce dependency on petroleum oils [24,25]. Surface texturing is an effective method to control the friction phenomenon without the need to use lubricants [26].

Understanding the friction conditions involves using an appropriate tribological test. So far, many methods have been developed to determine the value of the coefficient of friction (CoF) using experimental tests modeling contact conditions in specific areas of drawpieces [27]. This article focuses on determining the value of the coefficient of friction on drawbead, which is an element in the stamping die that introduces additional resistance to moving the sheet metal [28]. In this way, it is possible to equalize the material flow along the perimeter of the drawpiece or to introduce additional resistance in key areas of the formed sheet [29]. While passing through the drawbead, the sheet metal is bent and straightened many times. Determining the deformation resistance and the value of the CoF occurring on the drawbead is necessary to design the appropriate geometry of the drawbead and to plan the technological process of plastic processing. The finite element method (FEM) of numerical modeling is the main tool for analyzing the deformation of sheet metal in the drawbead region. Chabrand et al. [30] developed the finite element model to determine the main deformation paths in the drawbead zone. The results can be used for better approximations of the drawbead in FE-based simulation codes. Billade and Dahake [31] analyzed the thickness and strain variations during the forming process of the automotive component by using the finite element method. It was found that circular drawbeads are preferred over step beads. In the case of step beads, the sheet thinning is larger than circular beads. Desai and Deshmukh [32] optimized the draw bead location using the FEM for the panel header forming process. It was found that wrinkling tendency can be avoided by decreasing the entry side radius. Sena and Piyasin [33] applied the finite element method to optimize the drawbead pattern. The optimal drawbead shape chosen was the drawbead, which resulted in the least failure elements. Jung [34] analyzed the effects that the number of beads and the bead shape have on forming processes. Despite the high geometric nonlinearity of the problem, the authors did not observe a convergence problem.

The idea of determining the value of the CoF at the drawbead was discovered in 1978 by Nine [35], who proposed the use of a special tribological simulator, imitating the process of pulling a strip of sheet metal through a system of rollers. He found that metal deformation and friction contributions to the drawing and clamping forces for drawbeads can be separated based on Coulomb’s law. Triantafyllidis et al. [36] numerically investigated (finite element method) the effects of material properties, bead geometry and CoF on the force-displacement and strain distribution diagrams for several bead designs. They found that wide, shallow beads produce little risk of sheet tearing. On the other hand, deep, narrow beads provide the greatest restraining force during the pulling phase of deformation. The drawbead model proposed by Maker et al. [37] accurately predicts the effect of variations in bead geometry, material property and friction conditions on restraining force. In another article, Maker [38] optimized 3D drawbead designs through the finite element method, using the LS-Dyna software (Version 950, Livermore Software Technology Corporation, Livemore, CA, USA). Incorporating analytic drawbeads in 3D-forming analyses showed a promising outcome in the simulation of the product development cycle. Furthermore, Hance and Walters [39] investigated the effect of sheet thickness on the frictional forces using a drawbead simulator. They proposed a correction factor which normalizes the CoF over a range of sheet metal thicknesses. Leocata et al. [40] analyzed the change in the sheet topography caused by drawbead. It was found that the roughening due to plastic strain increases the boundary lubrication, and thus the CoF. Additionally, the surface roughness of the sheet metal increases during the drawbead pass due to plastic strain [41]. Ren et al. [42] established a drawbead model with linear Coulomb friction, and they obtained the restraining forces corresponding to a range of bead penetration depths. Figueiredo et al. [43] used the drawbead friction test to investigate the effect of the surface roughness, sliding speed and the lubrication regime on the CoF. The results revealed that the surface roughness of the die has a significant effect on the CoF.

Due to the complexity of the friction phenomenon, the synergistic influence of many parameters simultaneously on the value of the coefficient of friction or topography of the sheet surface caused by friction is difficult. Therefore, statistical analyses, artificial intelligence (AI) methods and machine learning (ML) are tools supporting tribological research. In regression, ML algorithms can be classified into the following groups: random forests, decision trees, support vector regression and linear regression and deep neural networks. Najm et al. [44] applied the CatBoost machine learning algorithm to identify the parameters of CoFs for carbon steel sheets tested in strip-drawing tests. CatBoost was able to predict the CoFs with *R^2^* values between approximately 0.95 and 0.97. Trzepieciński et al. [45] identified parameters affecting the CoF of 5000 series aluminium alloy sheets by using artificial neural networks. The authors investigated the influence of the activation function of neurons on the prediction accuracy of ANN. It was found that the leaky rectified linear unit was the most appropriate activation function. In paper [46], ANNs were used to model the influence of drawbead friction test parameters on the CoF. Four training algorithms (quasi-Newton, conjugate gradients, Levenberg–Marquardt and back propagation) were used to train the ANNs. The quasi-Newton algorithm was the most effective and provided *R*^2^ = 0.996 for the training set. Szewczyk et al. [47] used the CatBoost ML algorithm to estimate the mean surface roughness and CoF of DC05 steel sheets. It was found that contact pressure is the least significant factor in determining mean surface roughness, whereas the viscosity lubricant has no significant effect on CoF. Szpunar et al. [48] applied a split plot design and analysis of variance to obtain a response about the relationship between incremental forming process parameters and CoF. They obtained the relationships between the analyzed parameters using response surfaces of the model exhibited, wherein *R*^2^ = 0.96. The integration of analytical methods with tribology will inevitably improve the efficiency of analysis and research in tribology, and promote the development of component tribology [49], green tribology [50,51] and intelligent tribology [52,53]. Malinowski and Kasińska [54] presented a case study using ANNs and ML algorithms for the effective prediction of tribological properties of wear-resistant layers. It was found that using ML is possible to effectively determine the effect of individual tribological parameters on individually explained parameters, such as wear depth and wear area. ML regression models could be used to predict CoF by using input parameters such as chemical composition, microstructure, mechanical properties, coating deposition process and grain size [55]. Kchaou [55] proposed an innovative framework based on the coupling experiments and ML algorithm. The developed data-driven approach was used for the optimization of the experimental tribological test design and to discover correlations between friction process parameters (sliding distance, temperature, normal load, surface properties) and friction behavior. Cheng et al. [56] and Motamedi et al. [57] proposed a variation mode decomposition with ML algorithms to predict the CoF based on the surface roughness, friction noise and friction-induced vibrations. Baş and Karabacak [58] applied regression trees and support vector machine ML-based algorithms to model the effects of load and temperature on the CoF. Performance assessments demonstrated that analyzed models successfully predicted the value of the CoF. Noma et al. [59] used convolutional ANNs to recognize the relationship between the elemental distribution of a tribofilm and CoF. Gradient-weighted class activation mapping was developed to visualize the areas important for classifying elemental distributions into CoF groups; then, it was concluded that the CNNs are useful for evaluating friction of tribofilms formed from lubricant additives. Mohammed et al. [60] applied ANNs, Classification And Regression Tree (CART) and a support vector-supervised machine learning algorithm to predict the coefficient of friction of epoxy resin. Based on correlation coefficient *R*^2^ and the mean absolute error (MAE), it was shown that ANN had the best prediction ability. ML approaches allow for the discovery of correlations that are difficult to find using traditional methods. The K-nearest neighbor algorithm, principal component analysis and gradient-boosting machine algorithm have been successfully applied to the analysis of the frictional behavior of metallic composite materials [61]. Applications and the role of ML in tribology can be found in the review articles of Paturi et al. [62] and Sose et al. [63]. An overview of the applications of artificial intelligence (AI) in different tribological research fields, including intelligent tribology, and a basic theory of tribology can be found in paper [52].

To the best of the authors’ knowledge, this article uses gradient-boosting (GB) with a regression stress algorithm for the first time in the analysis of friction in sheet metal forming. Gradient-boosted decision trees involve implementing several models and aggregating their results. In gradient-boosting, the target of the functional space-boosting is pseudo-residuals, not the typical residuals used in traditional boosting. In the case of regression, the final result is generated from the average of all weak learners. In gradient-boosting, weak learners work sequentially by improving the successive analyzed models. This gives the model the advantage of predicting complex phenomena that are difficult to analyze with analytical methods, such as friction. This is a phenomenon that is influenced by many factors, which often interact synergistically. This makes it difficult to qualitatively assess the effect of individual friction conditions on the value of the coefficient of friction. The present study analyzes the influence of parameter features, specifically referred to as Features Importance, on the coefficient of friction of DC03 carbon steel, as determined in the drawbead friction test. A special tribometer was designed and manufactured to model the phenomenon of friction on the drawbead in the stamping die. The relative importance of the friction conditions (drawbead height, sample orientation and mean roughness) on the CoF was analyzed.

## 2. Material and Methods

### 2.1. Test Material

The test material was DC04 carbon steel sheet metal. The thickness of the sheets was 0.8 mm. Samples for friction tests had the form of sheet metal strips that were 400 mm long. Fejkiel [64] found that the width of the sheet metal strip determines the character of the sheet metal deformation and the value of the coefficient of friction in the drawbead test. Therefore, it was decided to test sheets of different widths, as follows: 7, 14 and 20 mm. Basic mechanical properties were determined for informational purposes using a uniaxial tensile test. This is the basic method for determining the mechanical properties of sheet metals. Tests using a Z100 (Zwick/Roell, Ulm, Germany) uniaxial testing machine were carried out in accordance with the EN ISO 6892-1:2020 [65] standard. The average values of basic mechanical properties for three different directions relative to the rolling direction (0°, 45° and 90°) (Table 1) were determined on the basis of three repetitions.

**Figure 1:**
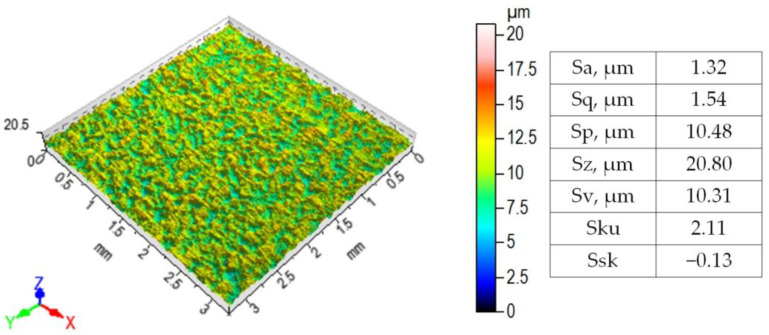
3D surface topography and selected roughness parameters of DC04 steel sheet.

Using the Talysurf CCI Lite profilometer (Taylor-Hobson Ltd., Leicester, UK), the sheet surface topographies were measured, and the basic surface roughness parameters (Figure 1) were determined. The profile analysis area was 3.25 × 3.25 mm^2^. The method of determining roughness parameters can be found in the ISO 25178-6 [66] standard.

### 2.2. Experimental

**Figure 2:**
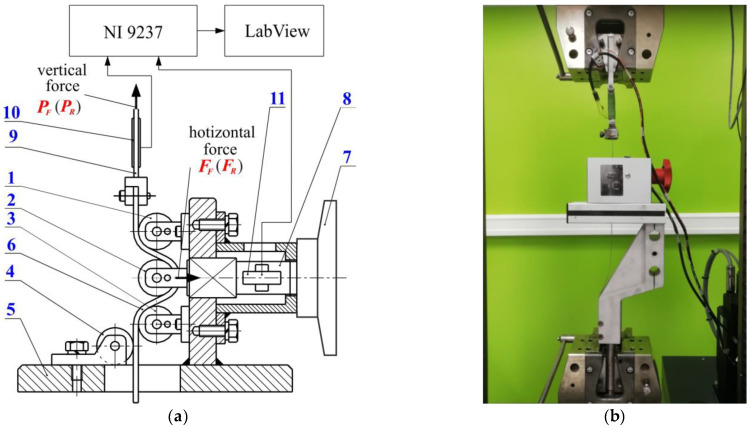
(**a**) Diagram and (**b**) view of the testing device: 1, 2, 3—working rollers; 4—support roller; 5—body; 6—sample; 7—nut; 8—horizontal tension cell; 9—upper tension cell; 10, 11—load cells.

Experimental tests were carried out on a specially designed device (Figure 2) whose operating principle was based on the Nine concept [35]. The device consists of a body, in which three cylindrical counter-samples are placed. The design of the tester enables the measurement of force parameters during the friction test. The test involves pulling sheet metal strips through a system of fixed rollers and through a system of freely rotating rollers. In this way, it is possible to separate resistance to friction from the resistance to plastic deformation of the sheet metal associated with repeated bending and straightening of the sheet metal during its passage through the drawbead. The device was mounted to the lower gripper of the Z100 universal testing machine (Zwick/Roell). One of the ends of the sample, in the form of a 400 mm long sheet metal strip, was mounted to the upper gripper of the testing machine.

**Figure 3:**
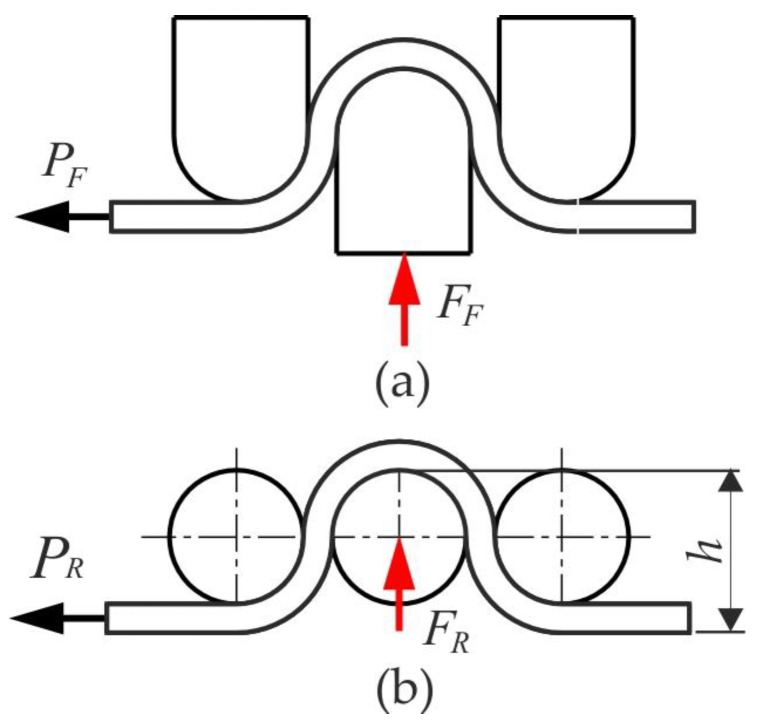
Scheme of force parameters for the test carried out with (**a**) fixed and (**b**) freely rotating rollers.

The value of the coefficient of friction is determined based on the values of horizontal and vertical forces that are recorded during tests with freely rotating and fixed rollers. Load cell sensors are placed on the horizontal tension cell and the upper tension cell (Figure 2a). During the test, the signal values from these sensors were recorded using the NI 9237 measurement card (National Instruments Xorporation, Austin, TX, USA) and the Lab View DAQ program (National Instruments Corporation, ver. 2015 SP1, Austin, TX, USA), with a frequency of 50 Hz. The diagram of the force parameters is shown in Figure 3. The tests were carried out for different heights of the drawbead *h,* as follows: 18 mm, 12 mm and 6 mm. The value of the coefficient of friction was determined from the following equation [67]:

(1) $$\mu =\frac{sin\alpha }{2\alpha }\times \frac{P_{F}-P_{R}}{F_{F}}$$

where α is the contact angle of the middle roller with the sample. *P_R_* and *P_F_* are the pulling forces obtained with the freely rotating rollers and fixed rollers, respectively; *F_F_* is the normal force obtained with fixed beads.

The change in the value of the coefficient friction was determined based on the recorded force parameters during the entire test of drawing the sheet metal through the drawbead. In this way, approximately 750 values of the coefficient of friction as a function of drawing distance *μ* = f (distance) were obtained for each test. Then, the average value of these approximately 750 values of the coefficient of friction was determined, which was representative for each test carried out under specific friction conditions.

The contact angle α values for the drawbead heights of 18 mm, 12 mm and 6 mm were 111.4°, 82.6° and 36.4°, respectively.

Rollers with a diameter of 20 mm are made of 145Cr6 tool steel. Three sets of rollers with different average surface roughnesses of 1.25 μm, 0.63 μm and 0.32 μm were used in the tests. The sliding speed was 10 mm/s. Three types of friction conditions were considered, as follows: dry friction and lubrication with oils with different kinematic viscosities, (η), Heavy-Draw 1150 oil (η = 1157 mm^2^/s) and LAN-46 machine oil (η = 43.9 mm^2^/s).

In summary, this section presents the experimental conditions for determining the coefficient of friction at the drawbead using a specially designed device. The test material was 0.8-mm-thick DC04 steel sheets. The second elements of the friction pair were countersamples made of 145Cr6 tool steel. Friction tests were carried out for the following variable parameters: sample orientation in relation to the sheet rolling direction, drawbead height and average surface roughness of the countersamples. The tests were carried out under dry friction conditions and under lubricated conditions with machine oil (LAN-46) and synthetic stamping oil (Heavy-Draw 1150). Based on the recorded force parameters of the test, the value of the coefficient of friction was determined according to Equation (1).

### 2.3. Gradient-Boosting Regression

Regression with boosted decision trees is used to establish the regression of friction process parameters on the predicted output of the coefficient of friction (COF), based on their impacts. The boosting technique involves sequential learning using previously fitted learners, as well as an analysis of errors [68]. In this study, the Gradient-Boosting Regression (GBR) algorithm in [69] and [70] is implemented for the prediction of COF. Decision trees are usually used with boost methods to enhance the prediction of shallow data. To improve the accuracy of the model, five MinLeafs were applied to the regression tree, together with 500 ensembles utilized for learning the previously learned nodes, noting that the MinLeaf indicates the minimum number of leaf node observations. In comparison to a tree leaf, each leaf contains at least one MinLeaf set of observations. The least-squares-boosting technique, often known as LSBoost, is used to train a group of regression trees. The LSBoost technique generates regression ensembles to minimize the mean-squared error (*MSE*). By finding the difference between the observed response and the summed error of the prediction of all learners, which have been trained in the previous step, fitting a new learner to the difference has been shown to be an effective method for minimizing the *MSE* [71].

### 2.4. Validation Metrics

The coefficient of determination (*R*^2^) and Standard Error Mean (SEM) were used to validate the COF prediction. SEM is achieved by dividing the initial standard deviation (SD) of a sample size by the square root of the sample size. *R*^2^ is the combined value of the Total Sum of Squares (*SS_tot_*) and the Sum of the Square of Residuals (*SS_res_*). The formulas to calculate the parameters mentioned above are as follows:

(2) $$E=\left(y_{i}^{target}-y_{i}^{predict}\right)$$

(3) $$MSE=\frac{1}{n}\sum_{i=1}^{n}{\left(y_{i}^{target}-y_{i}^{predict}\right)}^{2}\;\mathrm{or}\;MSE=\frac{1}{n}\sum_{i=1}^{n}{\left(E\right)}^{2}$$

(4) $$\bar{y}=\frac{1}{n}\sum_{i=1}^{n}\left(y_{i}^{target}\right)$$

(5) $${SS}_{tot}=\sum_{i=1}^{n}{\left(y_{i}^{target}-\bar{y}\right)}^{2}$$

(6) $${SS}_{res}=\sum_{i=1}^{n}{\left(y_{i}^{target}-y_{i}^{predict}\right)}^{2}\;\mathrm{or}\;{SS}_{res}=\sum_{i=1}^{n}{\left(E\right)}^{2}$$

(7) $$R^{2}=\frac{{SS}_{tot}-{SS}_{res}}{{SS}_{tot}}$$

Thus,

(8) $$R^{2}=\frac{\sum_{i=1}^{n}{\left(y_{i}^{target}-\bar{y}\right)}^{2}-\sum_{i=1}^{n}{\left(y_{i}^{target}-y_{i}^{predict}\right)}^{2}}{\sum_{i=1}^{n}{\left(y_{i}^{target}-\bar{y}\right)}^{2}}$$

where *E* is the error, $y_{i}^{target}$ are the actual values of COF, $y_{i}^{predict}\;$are the prediction values of COF, $\bar{y}$ is the mean value of the actual values of COF and *n* is number of measurements.

The Standard Error Mean (SEM) can be determined from the following equation:

(9) $$SD=\frac{SD}{\sqrt{n}}$$

where

(10) $$SD=\sqrt{\frac{1}{n-1}\sum_{i=1}^{n}{\left(\bar{E}-\bar{ME}\right)}^{2}}$$

(11) $$\bar{E}=\left(y_{i}^{predict}-y_{i}^{target}\right)$$

(12) $$\bar{ME}=\frac{1}{n}\sum_{i=1}^{n}\left(y_{i}^{predict}-y_{i}^{target}\right)$$

### 2.5. Categorical Variables

One-hot encoding is a commonly used method for expressing categorical variables, sometimes called dummy variables [68,72]. One-hot encoding is a technique that replaces a category variable with one or more additional features [73]. These inputs are effectively transformed into sparse-binarized representations by converting the category inputs into binary values of 0 and 1. These representations may subsequently be used as features to train artificial neural network (ANN) models. However, in this study, because the Gradient-Boosting Regression has been implemented in the CatBoost Jupyter Notebook, which supports executing categorical variables directly, the process of One-hot encoding has been skipped.

## 3. Results and Discussion

It is essential to distinguish between training and test errors while evaluating a model. Training errors are calculated using the same dataset to train the model, but a distinct, unseen dataset is used to calculate the test error. The *R*^2^ value of the training dataset quantifies the variation the model captures within the trained samples. On the other hand, the *R*^2^ value of the testing dataset acts as a measure of the model’s capacity to make predictions. However, the model was run after dividing the data (162 samples) into the training set and test set by allocating 80% of the actual data (129 samples) for training and storing 20% for testing, resulting in 33 samples. The reliability of the experimental data was verified by artificial neural network analysis. This analysis was presented in the previous article [46].

**Figure 4:**
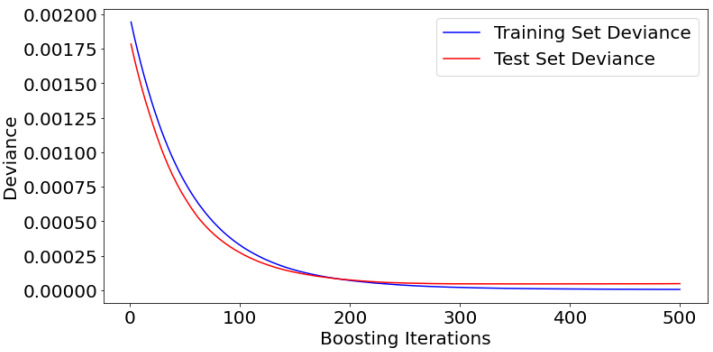
Model performance of the training and testing iterations.

As stated, 500 iterations were tuned to obtain the optimal *R*^2^ and minimum mean-squared error. Undoubtedly, augmenting the number of iterations may enhance accuracy, but at the cost of time, and there is a possibility of the model experiencing overfitting. Within this analysis, 500 iterations were conducted, which proved to be very effective in reaching optimal performance, as seen in Figure 4. Stability was achieved after around 300 iterations, with a slight decrease in *MSE* that is nearly insignificant in the final 200 iterations.

**Figure 5:**
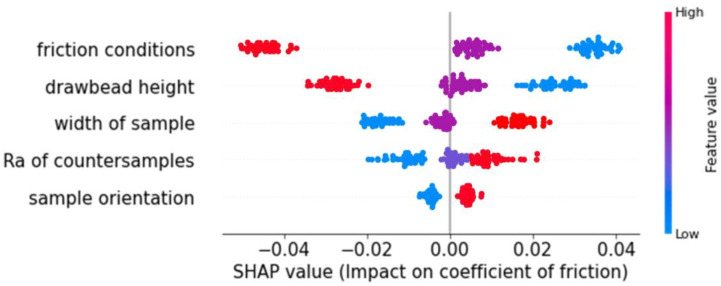
SHAP value plot influence on COF.

The SHapley Additive exPlanations (SHAP) plot shown in Figure 5 is regarded as a critical benefit of CatBoost in data analysis and evaluating the influence of inputs on outputs. There are several approaches to analyzing this effect; however, SHAP plots excel in scrutinizing each number within each group independently. Put simply, if the values of other parameters in the same row alter, the same value for a particular parameter may have a different impact than its previous impact, even if it has the same value. In Figure 5, the parameter of the sample width value is represented by the blue line on the far-left side. The last point on this line is positioned at −0.02. Without this value, the prediction would rise by 0.02. In contrast, excluding the number on the far-right in red, roughly 0.025, would result in a drop of 0.025 in the prediction. The divergence and convergence of data points result in fluctuations in the standard deviation, which in turn influence the average error and subsequently affect all validation metrics, thus affecting the performance of the model.

**Figure 6:**
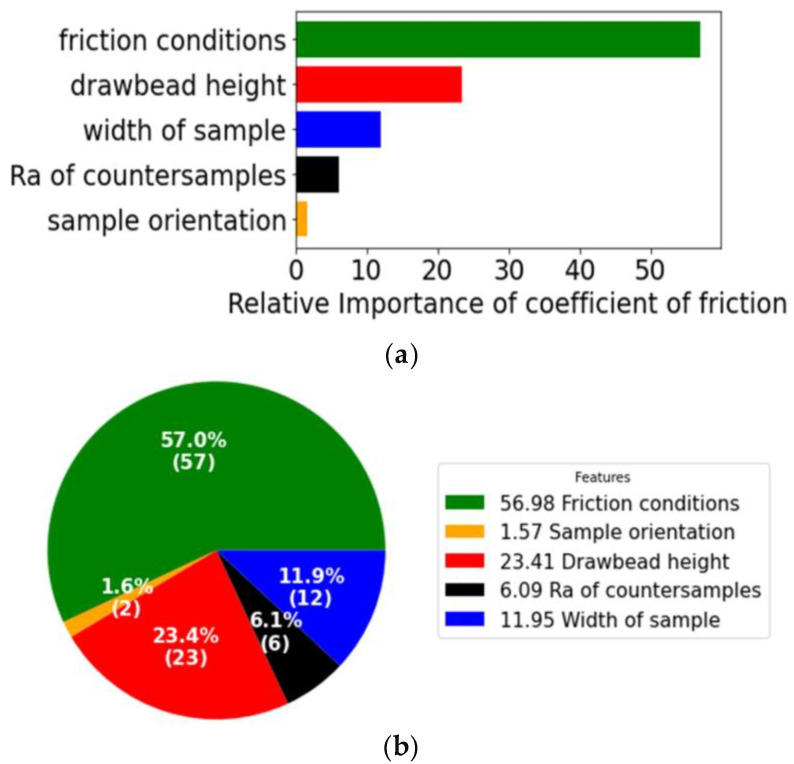
Relative importance of input parameters on COF; (**a**) ordered bar chart and (**b**) pie chart with percentage.

The present study analyzes the influence of parameter features, specifically referred to as Features Importance or relative importance, on the coefficient of friction, which is the output variable. As stated before, the experimental study included five parameters, which were employed as inputs. The Features Importance of the parameters is shown in Figure 6. The input parameter ‘Friction conditions’, as a categorical variable, is responsible for the lubrication conditions, i.e., it assumes one of the three following states: dry friction, lubrication using LAN-46 machine oil (Orlen Oil, Kraków, Poland) and lubrication using Heavy-Draw 1150 stamping oil (Lamson Oil, Rockford, IL, USA). The friction condition during experiments, consisting of three conditions (dry, using oil LAN-46 and using oil HD1150), had the most significant influence on the coefficient of friction (COF), followed by the drawbead height and the sample width. Lubrication conditions (dry friction or lubrication) play a key role in the phenomena occurring in the contact zone in sheet metal forming [74,75]. The use of liquid lubricants is a basic and effective way to reduce friction. Lubricant is used to separate rubbing surfaces by forming a continuous film at the contact interface. In this way, the mechanical cooperation of the surface asperities is limited, among others, through the mechanisms of flattening or ploughing [76]. Drawbead height determines the change in the topography of the sheet metal surface caused by repeated bending and straightening of the sheet when the sheet passes through the drawbead. Moreover, DC04 steel sheets have a high tendency to work hardening [77,78]. Therefore, large deformations of the sheet material change their strength and hardness at the same time. In this way, there is a change in the friction conditions, resulting from a change in the mechanical properties of one of the bodies of the friction pair. The width of the sample influences the character of the deformation of the sheet metal while passing through the drawbead. Therefore, there is a different intensity of frictional cooperation between the sheet and the countersamples across the width of the sheet strip [64]. This also affects the value and mutual relationship between the values of force parameters, which, according to Equation (1), are the basis for determining the value of the coefficient of friction.

The influence of the surface roughness of the countersamples was minimal. Lastly, the sample orientation had a minimal influence on the COF. Figure 6b displays the percentage of influence of several parameter features. The friction condition feature has the most influence, at 56.98%, followed by drawbead height, at 23.41%, sample width, at 11.95%, surface roughness of countersamples, at 6.09%, and sample orientation, at 1.57%.

As ‘friction conditions’ (Figure 6), the authors considered three friction process conditions, as follows: dry friction, lubrication using LAN-46 machine oil and Heavy-Draw 1150 stamping oil. During dry friction, severe plastic interaction of the tool and asperities of surface roughness of sheet metal occur. As a result of contact pressure, these surface asperities are flattened. This occurs in both dry friction and lubrication conditions. However, this phenomenon is more intense in dry friction conditions. In lubricated conditions, the friction surfaces are separated by a thin layer of lubricant, which limits adhesion phenomena in the contact zone, leading to a decrease in the CoF. ‘Drawbead height’ (Figure 6) determines the degree of sheet deformation during drawbead passage. Higher values of this parameter affect the greater degree of change in the sheet surface topography when strip sample-flowing through the drawbead. This is also associated with more intense work, hardening of the sheet material and a change in the mechanical properties of one of the materials of the friction pair (sheet metal). In these conditions, the change in the sheet metal surface topography limits the flattening mechanism. The ‘Width of sample’ (Figure 6) affects the character of sheet metal deformation when sample-flowing through the drawbead. Transverse components of stress arise when sheet metal is subjected to cyclic bending and straightening when passing through the drawbead, which distorts the process of deformation of the specimen along its width. This phenomenon is described in detail in the previous paper [79]. The average surface roughness of countersamples (‘Ra of countersamples’ in Figure 6) is responsible for the character of the friction pair cooperation. It should be noted that in sheet metal forming, the strength of the tool is greater than the strength of the sheet metal. The influence of the average surface roughness of countersamples depends on the friction conditions. During dry friction, the surface roughness of the tool affects the intensity of flattening and ploughing of the sheet metal surface. On the one hand, in lubricated conditions, the higher tool roughness provides a larger volume of valleys, which are a reservoir of lubricant. On the other hand, higher tool surface roughness increases the share of the ploughing phenomenon in total resistance to friction, thus increasing CoF. Cold-rolled steel sheets were used in the study. As a result of the production process (rolling), the sheet properties along the rolling direction and in the direction transverse to the rolling direction are different [79]. In this way, the deformation resistance of samples oriented in different directions, considered as ‘sample orientation’ (Figure 6), is different. As a result, the value of the friction process force parameters and the CoF, determined according to the relationship proposed by Nanayakkara et al. (Equation (1)) [67], changes according to sample orientation.

**Figure 7:**
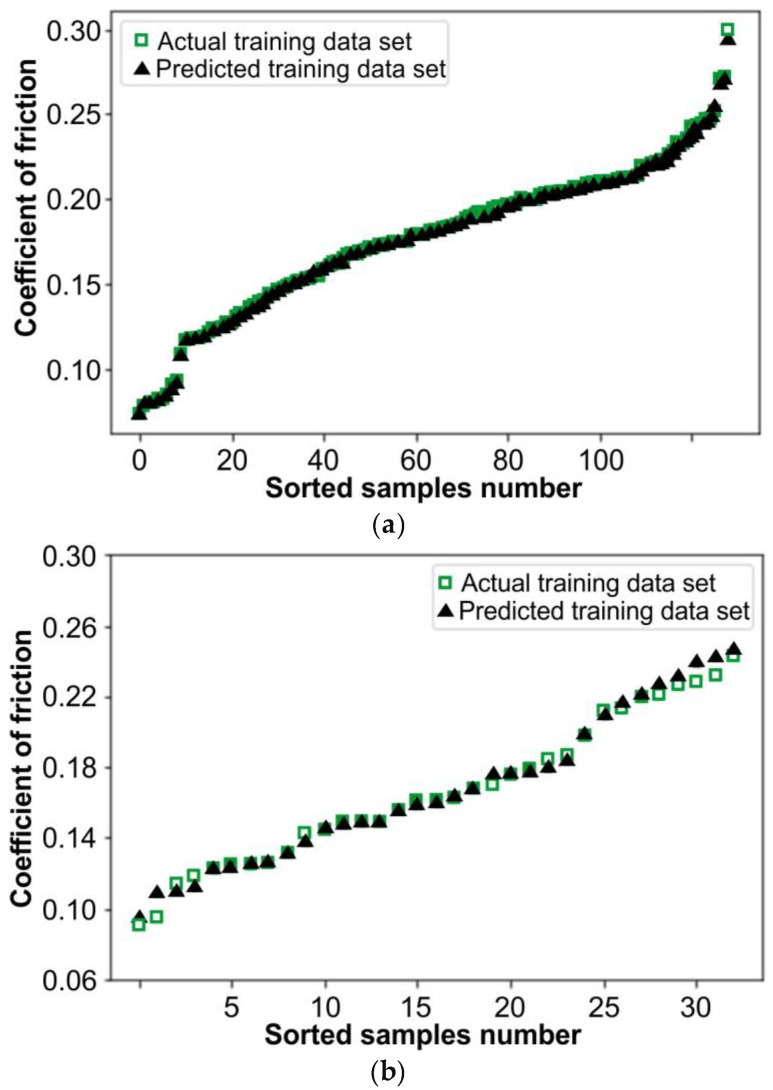
Actual and predicted values; (**a**) training COF dataset and (**b**) testing COF dataset.

**Figure 8:**
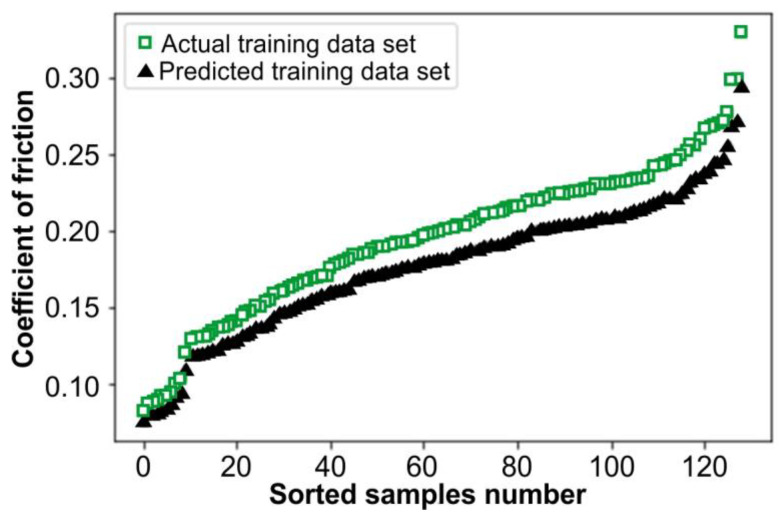
Actual and predicted values of training COF dataset with 0.1 adjusting.

Figure 7 illustrates the actual and predicted values of the COF. The *X*-axis shows the number of values, while the *Y*-axis reflects the value of the COF. The values are sorted in ascending order to prevent displaying the data in a skewed manner. However, it is not feasible to see the behavior of the predicted values if they align with the actual values and if they are not sorted. Figure 7a displays the actual and predicted COF values used for the training dataset of the model, whereas Figure 7b shows the data that were retained and not used in the training to be used for testing the model, known as the testing dataset. However, the data in Figure 8 correspond to the data in Figure 7a, with the exception that all the actual training values have been adjusted by 0.1 in the plot to differentiate between actual and predicted values. This adjustment was made because the predicted data in Figure 7a completely matched some of the actual training data, causing them not to be visible.

**Figure 9:**
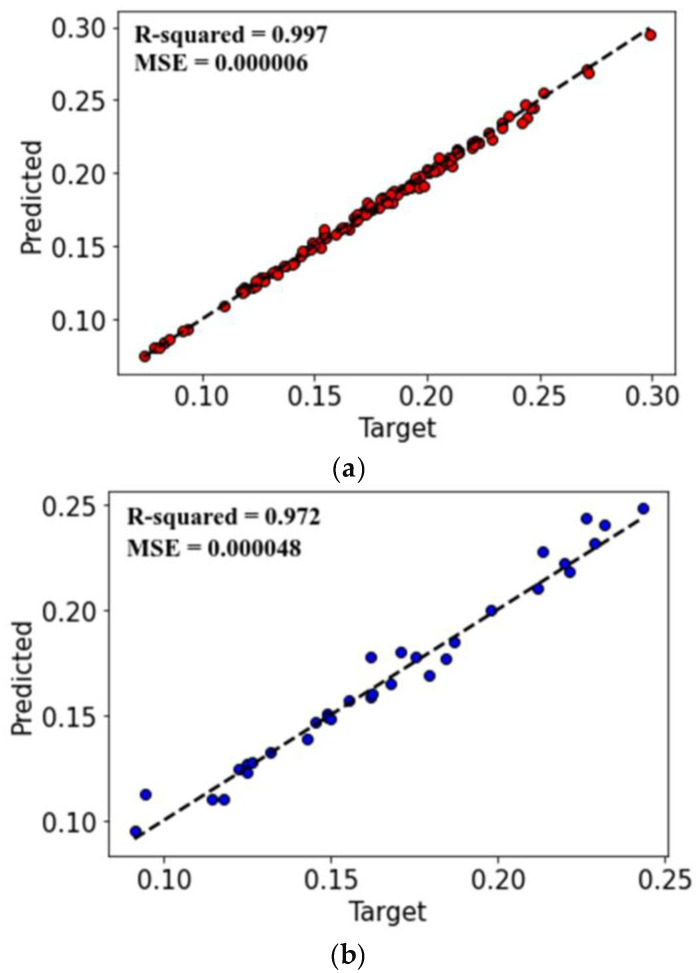
Actual and predicted values of COF; (**a**) training dataset and (**b**) testing dataset.

In Figure 9, the dashed line demonstrates an exact theoretical correspondence between the observed—actual data—and the predicted values of COF, with the data superimposed exactly on top of the line. The dispersion and deviation observed from the dashed line indicate the model’s ability to predict COF values with minimum errors. As mentioned earlier, the line represents the best fit, and the deviation of values from this line represents errors. Based on this, the standard deviation, mean-squared error and R-squared have been calculated using the equations referred to in the section of validation metrics.

Designing the sheet metal forming process with (or without) drawbeads requires knowledge of friction conditions. In recent years, the finite element method (FEM) has been increasingly used to model the forming process and its optimization. The numerical model requires knowledge of contact conditions in critical locations of the formed drawpiece. This paper presents an example of a tool design that can be used to determine boundary conditions at the drawbead in the FEM model. Analytical models for determining the coefficient of friction in this zone of the stamping die have not been developed yet. The Features Importance analysis, performed using the Gradient-Boosting with Regression Trees algorithm, provided information on the significance of selected friction process parameters. This allows the planning of future research to focus on the parameters most responsible for the friction phenomenon at the drawbead, omitting the least important ones. Due to the complex, difficult to analytically assess, synergistic effect of many parameters on the friction phenomenon, finding the most important factors influencing friction is difficult without statistical analysis.

## 4. Conclusions

This article presents an analysis of the impact of the importance of individual features on the coefficient of friction of DC04 steel sheets based on the experimental results of the drawbead test. The Gradient-Boosting with Regression Trees algorithm was used to analyze the experimental data. A group of regression trees was trained using the least-squares-boosting technique. Features Importance analysis has shown that the friction conditions (dry friction of lubricated conditions) had the most significant influence on the coefficient of friction, followed by the drawbead height and the sample width. The friction condition feature has the most influence on the coefficient of friction, at 56.98%, followed by drawbead height, at 23.41%, and sample width, at 11.95%. The average surface roughness of rollers and sample orientation have a small impact on the value of the coefficient of friction at 6.09% and 1.57%, respectively. The dispersion and deviation observed from the experimental data indicate the model’s ability to predict values of the coefficient of friction. The coefficients of determination for training and testing the datasets were *R*^2^ = 0.997 and *R*^2^ = 0.972, respectively.

Future research will test the predictive potential of the developed model for data that were not used in either the training set or the testing set. The extension of the experimental campaign to sheets of different roughness will allow for the representation in the data to have an effect on the interaction between the surface roughness of the tool and the sheet metal, and on the value of the coefficient of friction. The implementation of the investigations with the participation of oils with viscosity varying in a wide range would allow for the inclusion of lubricant viscosity in the gradient-boosting-based model. It will also be interesting to apply and compare other data processing algorithms, including machine learning, CatBoost (Yandex), LightGBM, eXtreme Gradient-Boosting (XGBoost), etc. The research presented in this article confirmed the effectiveness of experimental data to obtain a high-performance gradient-boosting-based model.

## Figures and Tables

**Table 1:** Basic mechanical properties of DC04 sheet metal.

Sample Orientation	Yield Stress, MPa	Ultimate Tensile Strength, MPa	Elongation, %
0°	184	304	23
45°	194	315	22
90°	176	296	23

## Data Availability

The raw data supporting the conclusions of this article will be made available by the authors on request.
